# Right ventricular outflow reconstruction with handmade valve conduit - A short experience from a developing country. Case series

**DOI:** 10.1016/j.amsu.2020.08.023

**Published:** 2020-09-01

**Authors:** Yasir Khan, Syed Shahabuddin, Muneer Amanullah

**Affiliations:** Section of Cardiothoracic, Department of Surgery, Aga Khan University Hospital, Karachi, Pakistan

**Keywords:** Handmade valve conduit, Cost effective, Developing country

## Abstract

**Objectives:**

Right ventricular outflow tract continuity abnormalities are one of the most commonly encountered entities in the field of congenital cardiac surgery. Various strategies including homograft, valve conduit, Contegra are used to restore continuity between right ventricle and pulmonary artery. In countries like Pakistan these may not be easily available and affordable. We report the experience of our short observational study of using a handmade trileaflet valve conduit to reconstruct the right ventricular outflow tract.

**Methodology:**

From September 2015 to December 2016, a total of 15 patients with different congenital heart diseases underwent open-heart surgery at our institute. Restoration of right ventricular to pulmonary artery continuity was achieved using handmade valve conduit utilizing bovine pericardium and thin sheet PTFE sheets (0.1 mm) as conduit and valve respectively.

**Results:**

Patients ranged from 1 to 16 years. Seven patients had previous palliation including 4 blalock taussig (BT) Shunts and 3 pulmonary artery (PA) banding. Postoperative complications were observed in 4 patient including 2 in hospital deaths and 2 required interventions. One patient developed aneurysm at RV- conduit junction requiring surgical repair and the other underwent conduit dilatation for moderate to severe stenosis (gradient 60 mmHg). No significant regurgitation was observed in this series. Overall postoperative gradients were stable with mean gradient 25.3 mmHg (8 mmhg - 60 mmHg).

**Conclusion:**

The use of handmade valve conduits has acceptable morbidity and mortality. These are cost effective alternatives in this part of the world, where well-established conduits have cost implications and uncertain availability.

## Highlights

• Reconstruction of Right ventricular to Outflow tract is challenging surgically.
• Right ventricular outflow tract reconstruction using bovine pericardium and PTFE graft is cost effective
• Right Ventricular outflow tract reconstruction using bovine pericardium and PTFE has reasonable outcome.

## 1 Introduction

The following case series has been reported from Our University Hospital which is an internationally recognized teaching hospital and a tertiary care center based in Pakistan, in accordance with the PROCESS guidelines for case series [24]. Right ventricle–pulmonary artery (RV-PA) anatomic discontinuity is present in approximately 5% of hearts with congenital defects [1]. Some of the conditions that need restoration of continuity are variants of tetralogy of Fallot (TOF), pulmonary atresia and in discordant heart morphology [2]. Various strategies including homograft, valve conduit, bovine internal jugular vein (Contegra), and patch enlargement with valve replacement or reconstruction are used to restore anatomical and functional continuity between right ventricle and pulmonary artery [3]. It is important to note that these children do need a repeat operation as either they grow over fixed conduit or conduit needs replacement because of structural degeneration [4]. These conduits are readily available in affluent nations. However in most of the developing countries the option of homograft is nonexistent or plenty of resources are needed to acquire them [5]. Similarly synthetic valve conduits and Contegra have cost implications as well as sometimes questionable availability. Trileaflet valve reconstructed with Polytetrafluoroethylene (PTFE) has been used as cost effective alternative and has shown promising short term and long term outcome [6] and requires minimal expertise to learn the technique of reconstruction [7]. In developing country cost is of paramount importance and implication of such cost effective procedure can help in operating on those congenital patient that were previously inoperable because of cost and unavailability of conduit. We report the experience of our short observational study of using a handmade trileaflet valve conduit using bovine pericardium and PTFE to reconstruct the right ventricular outflow tract from a developing country.

## 2 Materials and methods

This is a retrospective descriptive study. The study is conducted at the Aga Khan University Hospital; it is a multidisciplinary tertiary care hospital. All those patients where hand made trileaflet valve conduit was constructed and used are included. The patients receiving synthetic valve conduit or contegra (bovine jugular vein) were excluded. From September 2015 to December 2016, a total of 15 consecutive patients with different congenital heart disease diagnosed in the pediatric age group (up to 16 years) at our institute, the Aga Khan University Hospital underwent open-heart surgery with restoration of RV to PA continuity by using handmade valve conduit. The size of the conduit was determined by using an available nomogram. The data was collected from medical records regarding the demography, past medical history, surgical management and postoperative follow ups. The confidentiality and anonymity of the participants is ensured. The data was collected through charts and kept confidential and available only to the research participants.

### 2.1 Surgical stratigies

**Fig. 1:**
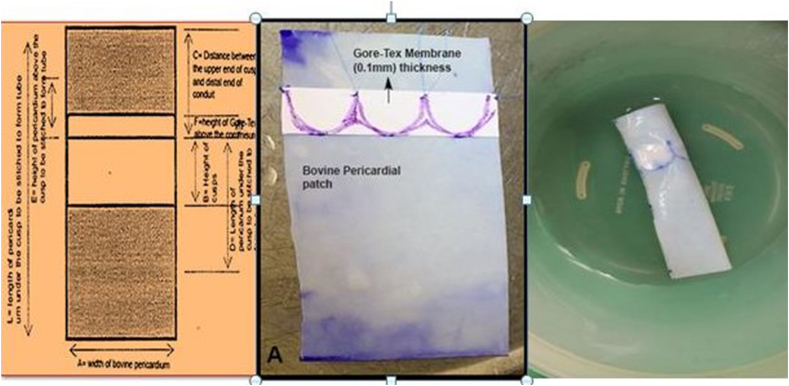
Shows constructed PTFE valve inside Bovine pericardium conduit.

**Fig. 2:**
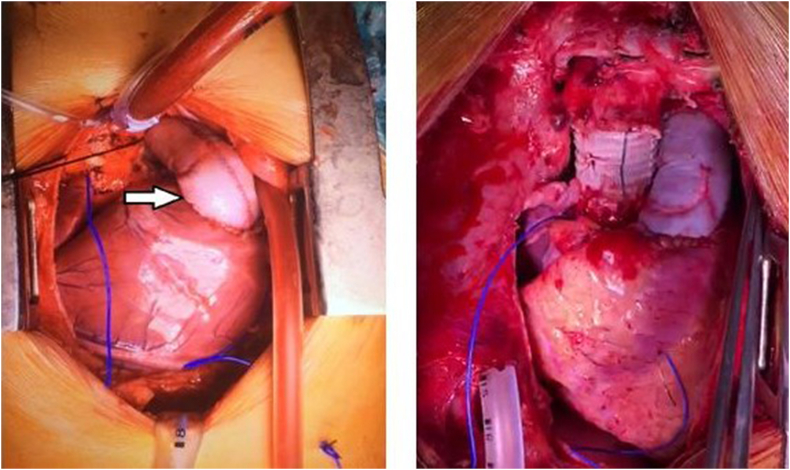
Intraoperative picture of right ventricular outflow tract reconstruction with handmade valve conduit.

All the procedures were performed by the same group of experienced and qualified surgeons. The two surgical teams worked simultaneously with one team operating on the patient and the other helped in fashioning of the valve conduit so as to reduce the overall operating time. The valve conduit was constructed according to the nomogram as determined by the size of the patient. The bovine pericardium and thin sheet PTFE sheets (0.1 mm) were used to construct conduit and valve respectively (Fig. 1). The operating room preparations were made as per hospital standardized protocol and the surgical procedure was performed in a routine fashion based on clinical diagnosis and indications on preoperative assessment. At the end of the surgical procedure, intraoperative echocardiography was performed to ensure adequacy of intracardiac repair and handmade valve conduit function (Fig. 2).

## 3 Results

A total of 15 patients underwent handmade trileaflet PTFE valve conduit right ventricular outflow tract reconstruction from 2015 to 2016. Age range from 1 to 16 year. Table 1 shows indications for surgery. There were four patients of Tetralogy of fellot with absent pulmonary valve. Pulmonary atresia with major aortopulmonary collateral arteries (MAPCAS) and truncus arteriosus were second most common indication with 3 patients in each group. Two patients had d transposition of great arteries (d TGA) with pulmonary stenosis and two patients underwent Ross procedure. One patient underwent 3rd time redo procedure for RV to PA homograft stenosis. Seven patients had previous palliation surgery including 4 Blalock-Taussig Shunts (BTS) and 3 Pulmonary Artery banding (PAB) (Table 2). One of the PAB was bilateral in a patient with Truncus.

**Table 1:** Indications for surgery.

Indications for surgery	Number of Patients
TOF with absent pulmonary valve	04
Pulmonary atresia with MAPCS	03
Truncus Arteriosus	03
dTGA with VSD and pulmonary stenosis	02
Ross	02
s/p RV-PA homograft conduit	01

**Table 2:** Previous interventions.

Previous interventions	Diagnosis at admission	Number of patients
BT shunt	dTGA + VSD + pulmonary stenosis	02
	TOF with absent pulmonary valve	02
PA banding	Truncus arteriosus	03

Table 3 shows the overall outcome. There were two in hospital deaths unrelated to conduit. One of the patient developed irreversible neurological dysfunction and in the due course died of multiorgan failure and second patient developed pulmonary hemorrhage as a result of coagulopathy. Major morbidities were observed in two patients. One patient developed pseudo aneurysm adjacent to valve conduit and right ventricular junction that was surgically repaired. The other patient gradually developed valve conduit stenosis with rising gradient (60 mmhg). This patient underwent percutaneous dilatation. No significant regurgitation was observed in any of the cases.

**Table 3:** Results.

Total number of patients	15
Age	1–16 years
Morbidity	02
Mortality	02

Overall postoperative gradients were stable with mean gradient 25.3 mmhg (8 mmhg – 60 mmhg) (Table 4). All the patients were followed from one to sixteen months with a mean follow up of 5.5 months.

**Table 4:**
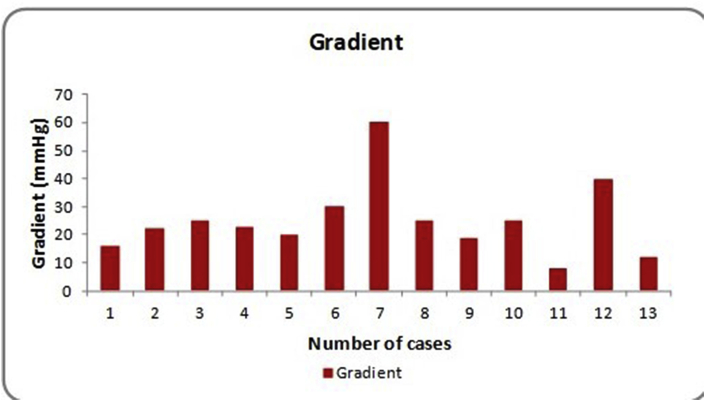
Gradient.

## 4 Discussions

The idea of using a valve conduit for better hemodynamic performance has made it possible to treat several complex congenital disorders that would not have been possible otherwise [8]. Use of an extra cardiac conduit between the right ventricle and the pulmonary arteries has made possible the routine repair of pulmonary atresia [9] complex tetralogy of Fallot [10], truncus arteriosus [11], transposition of the great arteries with ventricular septal defect and pulmonary stenosis [12], and other complex forms of congenital heart disease [13]. Similarly in patients who undergo Ross operation the pulmonary valve is replaced with an extra cardiac conduit [2]. The ideal choice for a valve conduit, however, has yet to be found. Desired characteristics include availability, ease of implantation, and longevity. Different substitutes have been proposed including fresh aortic homograft [14], Dacron valve conduits containing bioprostheses [15] or mechanical valves, more recently cryopreserved aortic or pulmonary homografts [16] and bovine jugular vein [17].

Homografts were the initial valve conduit used for reconstruction of the right ventricular outflow tract and was first reported for pulmonary atresia in 1966 [8]. It's the most widely used conduit in the United States. Although the initial result of homograft were excellent in term of mortality [18] but certain disadvantages exist such as such as lack of sufficient availability, the requirement of sterilization and preservation, and late complications due to degenerative processes and calcifications [6]. These limitations are for those where it is at least available. We in fact face huge problem of acquisition of these homograft even if we ignore the associated high cost.

Due to early graft failure of homograft, porcine heterografts mounted in Dacron conduits became available in a variety of sizes and were widely used for complex reconstructions [8]. The results were disappointing, characterized by poor handling, excessive intimal fibrocalcific peel formation, and degeneration of the porcine valve—particularly when implanted in children [19].

The lack of small-size homograft availability, overall scarcity and the concerns about their long-term outcome [20] have led to the development Contegra valve bovine conduit [21]. Contegra graft showed promising early and late performance however it was associated with distal conduit stenosis, significant graft dilatation and risk of thromboembolism [5]. One of the major concerns is lack of timely availability in required sizes in our part of the world. Above all it also has significant cost implications.

Mortality has never been a consideration related to restoration of RV to PA continuity with a conduit and the studies suggest that the use of contegra or other types of valve conduits were not directly contributing to early deaths as no technical problem related to conduit placement was identified and in fact, even on autopsy anatomical integrity was intact [21,22]. We also have similar observation in our study where two patients died of reason other than the interposition of valve conduit, where one of the patients died of pulmonary hemorrhage and other developed irreversible neurological dysfunction.

Despite advances in surgical technique and new conduit types, most RV-PA conduits will require revision in due time. Thus, patients who survive to adulthood face the prospect of >1 conduit replacement in their lifetimes [4].

The requirement of redo surgery with cost implications and unavailability of majority of conduit has led to development of right ventricular outflow tract reconstruction by using a PTFE sheet (of various thicknesses). The PTFE sheets for leaflet reconstruction are inexpensive and easily available. It has unique property of very low tissue affinity. Because of this property, cellular or fibrinous deposition is unlikely, and for these reasons, it is the most reliable material for small-caliber vascular grafts [5]. Also because of its chemical inertness, tissue degeneration or destruction is unlikely or very slow to progress. Long term result have shown promising outcome with handmade PTFE graft [22].

In a country like Pakistan where cost is an extremely important prohibitive factor in carrying out such complex procedures where valve conduits are required and repeated procedures are anticipated, leading to immense financial burden on health care. We have started constructing handmade trileaflet valve conduit in the operating room using bovine pericardium and a PTFE sheet according to the size of the patient using a nomogram established for this purpose (Fig. 1, Fig. 2). Bovine pericardium is used to construct tube graft and for leaflet reconstruction PTFE sheets are used. Previous studies [5] have used Dacron for tube formation and PTFE for leaflet construction. Zhang et al., used PTFE for tube graft formation along with trileaflet reconstruction. Both Dacron and PTFE for tube formation are costly than bovine pericardium [23]. Using this technique it has helped us to overcome cost and the shortage of commercially available valve conduits.

Our Result of handmade valve conduit were encouraging and for developing countries like Pakistan where there is unavailability of majority of conduit and cost issue for homograft, Contegra (2500 $) and synthetic valve conduit, Handmade trileaft PTFE valve conduit (700$) is cost effective and excellent alternative with desirable result.

The study is limited by the fact that number of patient is too small to justify statistical testing for a meaningful significance. The other limitations are the retrospective nature of study and that only short term results are reported. The strength of this modest study is that this will guide to resolve the problems associated with excessive cost and the lack of availability of homograft, synthetic graft and contegra. The technique of reconstruction is simple and reproducible and the material used is cost effective and has much better availability in this region.

## 5 Conclusions

Handmade trileaflet valve conduit using bovine pericardium and PTFE provides reliable alternative for RVOT reconstruction yielding to its cost effectiveness and easy availability especially in developing countries with good short term outcome.

## Sources of funding

None.

## Ethical approval

Department exemption taken.

## Author contribution

**Yasir Khan:** Data collection, manuscript writing, statistical analysis

**Syed Shahab:** Statistical Analysis, Manuscript editing.

**Muneer Amanullah**: final analysis.

## Research registration number

1. Name of the registry: Research Registry.

2. Unique Identifying number or registration ID: researchregistry5703.

3. Hyperlink to your specific registration (must be publicly accessible and will be checked): : https://www.researchregistry.com/browse-the-registry#home/.

## Guarantor

Syed Shahab

Assistant Prof, Cardiac Surgery

Aga Khan University Hospital Karachi, Pakistan.

## Consent

Informed consent taken from all patients.

## Provenance and peer review

Not commissioned, externally peer reviewed.
